# Strengthening Libya's pandemic preparedness using the National Bridging Workshop One Health Roadmap

**DOI:** 10.1016/j.onehlt.2026.101550

**Published:** 2026-08-19

**Authors:** Asma Saidouni, Ramy Mohamed Ghazy, Ramdhan Mohamed Osman, Abdulaziz Zorgani, Omar Elahmer, Hebatalla Abdelmaksoud Abdelmonsef Ahmed, Ahmed Elgrari, Guillaume Belot, Reem Ayad, Hatem Meslati, Iman Ben Hamza, Hayfa Abraheem Mohammed Alkourbu, Salem Abudher, Taher Emahbes, Maria Grazia Dente, Eszter Horvath, Haider Elsaeh, Ahmed Zouiten

**Affiliations:** a World Health Organization, Libya; b Tropical Health Department, High Institute of Public Health, Alexandria University, Alexandria, Egypt; c Family and Community Medicine Department, College of Medicine, King Khalid University, Abha, Saudi Arabia; d Faculty of Medical Technology, Department of public Health, University of Tripoli, Libya; e Libyan Centre for Disease Control, Tripoli, Libya; f Department of Medical Microbiology, Faculty of Medicine, University of Tripoli, Tripoli, Libya; g Faculty of Medical Technology, Department of Dental Technology, University of Tripoli, Tripoli, Libya; h Public Health & Community Medicine, Faculty of Medicine, Kafr-Elsheikh University, Kafr-Elsheikh, Egypt; i Department of Medical Parasitology, Faculty of Medicine, University of Tripoli, Tripoli, Libya; j World Health Organization, Geneva, Switzerland; k World Health Organization, Regional Office for the Eastern Mediterranean, Cairo, Egypt; l National Centre for Animal Health, Tripoli, Libya; m Ministry of Environment, Tripoli, Libya; n Ministry of Local Government, Tripoli, Libya; o Food and Drug Control Center, Tripoli, Libya; p National Center for Global Health, Italian Institute for Health, Rome, Italy; q Voluntas, Denmark

**Keywords:** Health security, One Health, Multisectoral collaboration, Zoonotic disease, Antimicrobial resistance, Health system preparedness

## Abstract

**Background:**

Libya faces recurrent and emerging health threats at the human, animal, and environment interface, compounded by prolonged instability and fragmented health systems. The National Bridging Workshop (NBW) is a structured approach to identify multisectoral collaboration gaps and develop a joint One Health Roadmap aligned with international health security frameworks. This study aimed to describe Libya's first NBW and evaluate its effect on increasing participants' One Healthknowledge.

**Methods:**

We conducted a quasi-experimental study using a single-group longitudinal design with pre-intervention, immediate post-intervention, and three-month follow-up assessments of One Health knowledge. The intervention was to deliver the NBW in Tripoli from 23 to 25 February 2025. Over three days, multisectoral stakeholders assessed collaboration across 16 technical areas using four locally adapted hazard scenarios (rabies, leishmaniasis, salmonellosis, and antimicrobial resistance [AMR]), mapped priority gaps to relevant International Health Regulations and Performance of Veterinary Services (PVS) indicators and co-developed a One Health Roadmap.

**Results:**

Seventy participants from human health, animal health, environmental health, food and drug control, and academia attended. Collaboration gaps were most consistently observed in technical-level coordination, sustainable financing, laboratory systems, education and training, and emergency funding across scenarios. Participants developed a joint roadmap comprising 11 objectives and 26 activities, with sustainable financing and the integration of One Health into legislation identified as the key priorities. Knowledge scores differed significantly across the three assessments time points (Friedman test, *p* < 0.001; Kendall's W = 0.44), indicating a moderate overall effect. Pairwise comparisons showed significantly higher scores immediately after the workshop than at baseline (median, 19 (18–20) vs. 16; *p* < 0.001; rank-biserial correlation, *r* = 0.64) and at three months than at baseline (median, 18.5 (15–20) vs. 16 (13–18); *p* = 0.015; *r* = 0.40).

**Conclusions:**

The NBW was successfully implemented in a fragile setting and generated a prioritized One Health Roadmap. Knowledge gains that persisted at three months suggest that NBW may contribute to capacity building beyond planning outputs, supporting the longer-term operationalization of One Health and strengthening preparedness and response.

## 1 Introduction

Libya is highly susceptible to a range of complex health threats, including zoonotic and vector-borne diseases, food-safety concerns, and the growing threat of antimicrobial resistance (AMR), alongside the effects of climate change and environmental degradation [1]. These challenges are intensified by ongoing political instability, armed conflict, and a fragile healthcare system, while Libya's geographic proximity to several African countries further heightens its vulnerability to transboundary health risks [1], [2]. Against this background, the One Health approach is central to addressing challenges that sit at the intersection of human, animal, and environmental health. It emphasizes the engagement of all relevant stakeholders and provides a basis for multisectoral collaboration, most recently through the formalized memorandum of understanding (MoU) that institutionalized the One Health approach in Libya [1], [2], [3], [4], [5], [6].

Recent work in Libya has sought to leverage existing capacities to address endemic and emerging zoonotic threats and to identify critical gaps [7]. Two further milestones were the identification of priority zoonotic diseases in Libya and of key transboundary zoonotic diseases, intended to improve timely information sharing and coordination both within Libya and with Tunisia [6]. Nonetheless, effective multisectoral collaboration remains challenging in several areas, including joint leadership and coordination, information sharing, and epidemiological functions such as surveillance and risk assessment, as well as resource mobilization for shared responsibility across the human, animal, and environmental health sectors in the fight against zoonotic diseases [8].

The International Health Regulations (IHR) constitute a legally binding framework that uses the World Health Organization (WHO) Monitoring and Evaluation Framework (MEF) to strengthen national capacities [9]. The IHR provide tools to assess core capacities, including the States Parties Self-Assessment Annual Report (SPAR) and the Joint External Evaluation (JEE) [10], [11]. Libya received an overall score of 25.3 out of 100 on the Global Health Security (GHS) Index, ranking 172 among 195 countries. The 2018 JEE reported scores below 40, highlighting limited preparedness to manage public health emergencies, particularly those involving infectious diseases [12], [13]. Complementing this human-health-focused framework, the Performance of Veterinary Services (PVS) Pathway is a global framework for the sustainable improvement of national veterinary services. The initial PVS evaluation took place in 2009, and a 2013 gap analysis report provided detailed findings and recommendations aligned with the standards of the World Organization for Animal Health (WOAH) to protect public health, safeguard livestock, and contribute to regional and global health security [13], [14], [15].

The National Bridging Workshop (NBW) uses these assessment frameworks (JEE and PVS) to find obstacles and gaps in intersectoral collaboration and to develop a joint roadmap for strengthening cooperation across sectors. As of 2025, more than 60 countries had conducted an NBW [16]. Countries such as Somalia, Kenya, Cameroon, and Thailand have used the NBW to establish joint roadmaps and stronger multisectoral linkages for a more robust One Health response to health threats [17], [18], [19]. Building on its 2009 PVS evaluation and 2018 JEE, Libya used the NBW as a structured platform for the human, animal, and environmental health sectors to identify deficiencies in multisectoral collaboration across critical technical areas and to co-develop a comprehensive One Health Roadmap.

This study, therefore, had two complementary objectives. The primary objective was to describe the implementation of Libya's first NBW and the participatory development of a prioritized One Health roadmap to strengthen multisectoral collaboration. The secondary objective was to evaluate, using a quasi-experimental one-group pretest–posttest design, whether the standardized educational components delivered during the NBW workshop were associated with measurable changes in participants' One Health knowledge over time.

## 2 Methods

### 2.1 Study design

We conducted a quasi-experimental study using a single-group longitudinal design with pre-intervention, immediate post-intervention, and three-month follow-up assessments. The study evaluated the integrated One Health educational intervention delivered during the NBW. Because no control group was available, the observed changes in knowledge cannot be attributed exclusively to the workshop. The three-day workshop took place in Tripoli, Libya, from 23 to 25 February 2025, and was conducted back-to-back with a One Health situation analysis undertaken in collaboration with the Istituto Superiore di Sanità (ISS). The workshop simultaneously served as the planning platform for developing the One Health Roadmap and the learning intervention being evaluated.

### 2.2 Participants

The study population comprised all workshop attendees, representing the human, animal, and environmental health sectors. A total of 70 participants attended, representing central, sub-national, and district levels of major national institutions, including the Ministry of Health (MoH), Ministry of Agriculture (MoA), Ministry of Environment (MoE), Ministry of Local Government (MoLG), Food and Drug Control Center (FDCC), National Center for Animal Health (NCAH), National Center for Disease Control (NCDC), and academic institutions. Participants included 28 representatives from public health, 11 from animal health, 19 from environmental health and local sanitation services, 6 from food and drug regulation, 5 from academia and 1 from WHO. The group was selected from both national and decentralized levels to ensure representation from all regions of the country and to cover a wide range of technical disciplines, including surveillance and response, laboratory services, infection prevention and control (IPC), AMR, zoonotic and vector-borne diseases, food safety, environmental health, climate change, points of entry, wildlife health, and risk communication and community engagement (RCCE). Invitations were coordinated through the IHR National Focal Point. Owing to security constraints, the workshop was facilitated by a lead WHO Libya office facilitator, with support from a trained facilitator from ISS. All participants received an Arabic-translated handbook outlining the workshop objectives, session instructions, and expected outputs. Each was assigned a unique identification number to preserve anonymity while enabling longitudinal assessment of learning outcomes.

### 2.3 Intervention: The National Bridging Workshop and its educational components

#### 2.3.1 Pre-workshop planning

Following the signing of the national One Health MoU in November 2024 [1], each relevant sector nominated a One Health focal point. After an official request to conduct an NBW in Libya, the WHO Country Office convened preparatory meetings with focal points from the MoH, MoA, MoE, MoLG, NCDC, NCAH, Environmental Sanitation Affairs (ESA), and FDCC. These meetings familiarized stakeholders with the NBW methodology, established the agenda, defined participant selection criteria to ensure multisectoral, multidisciplinary, and geographical representation, and jointly developed the case scenarios used for the collaboration gap assessment, with an emphasis on national ownership. Following consultation, rabies, leishmaniasis, salmonellosis, and AMR were selected as case studies to reflect a broad range of threats at the human–animal–environment interface, different transmission pathways, and varying opportunities for cross-sectoral collaboration. Rabies and leishmaniasis were prioritized for their public health importance as zoonotic diseases in Libya [3]. Rabies highlighted animal population management and waste disposal, while leishmaniasis highlighted vector control and environmental determinants. AMR was included as a high-priority threat identified in the 2024 Strategic Tool for Assessing Risks (STAR) profile, and salmonellosis was selected as a foodborne scenario to assess intersectoral coordination during food-safety incidents.

#### 2.3.2 Workshop conduct

The workshop followed the standardized NBW methodology and comprised seven structured sessions that guided participants from gap identification to the development and prioritization of a joint One Health Roadmap. The opening session introduced the objectives and national mandates and established a shared understanding of One Health. The MoH highlighted IPC and AMR strategies; the NCDC outlined surveillance and response plans for zoonotic and arboviral diseases; the NCAH emphasized disease prevention and food safety; and the MoE detailed regulatory roles in climate change, environmental pollution and pesticide management. International collaboration was showcased through the ISS–WHO partnership that supported the operationalization of One Health in Libya. Participants then used the four case scenarios to assess collaboration across 16 technical areas, mapped the identified gaps onto the IHR–PVS matrix, reviewed findings from the JEE and PVS evaluations, and formulated objectives and activities that were consolidated and prioritized into a single roadmap during the closing session. A detailed, session-by-session description of the seven sessions is provided in Supplementary File 1.

#### 2.3.3 Educational components

The educational intervention consisted of standardized training components embedded within the NBW methodology, including instructional videos on One Health collaboration, the IHR and evaluation frameworks, and environmental health tools. Participants also took part in facilitated small-group discussions using outbreak- and risk-based scenarios to apply One Health principles in identifying risk drivers, transmission pathways, and multisectoral control and response measures.

### 2.4 Data collection

Participants' knowledge was assessed at three points: before the workshop (pre-intervention), immediately afterwards (post-intervention), and three months after completion (follow-up). The unique identification numbers assigned to participants preserved anonymity while enabling longitudinal comparisons across the three phases.

Knowledge was measured using a structured questionnaire developed specifically for this study to cover seven thematic domains aligned with the workshop's learning objectives: core One Health concepts; zoonotic diseases; AMR; environmental and climate change impacts on health; multisectoral collaboration; global health security frameworks; and One Health governance. The instrument comprised 20 items in multiple-choice and true/false format, with items adapted from standardized NBW One Health training materials. Each item was scored as correct (1) or incorrect (0), giving a total score ranging from 0 to 20, with higher scores indicating greater One Health knowledge. Content validity was assessed through expert review by WHO specialists and experts in public health, One Health, Zoonoses, risk assessment, outbreak investigation, and climate change. Reviewers assessed the relevance and clarity of each item and its coverage of the seven domains. The questionnaire was then piloted with five professionals across sectors and disciplines who did not attend the workshop to assess clarity, comprehensibility, and completion time, leading to minor wording refinement. We did not formally evaluate internal consistency reliability because the questionnaire was created specifically for this workshop and was mainly designed to measure knowledge across separate thematic domains. The final English-language questionnaire is provided as Supplementary File 2.

The same questionnaire was administered immediately after the workshop and again three months later to assess changes in knowledge over time. Participants were not informed in advance of the delayed post-test to minimize the likelihood of advance preparation and testing effects. The three-month questionnaire was disseminated via a dedicated workshop communication platform.

### 2.5 Statistical analysis

Data was extracted into Microsoft Excel and analyzed using Statistical Package for Social Sciences (SPSS), version 27 (IBM Corp., Armonk, NY, USA). Before data analysis, the dataset underwent quality assurance and cleaning, including checks for completeness and consistency, and identification of outliers. Categorical variables were summarized as frequencies and percentages. Continuous variables were tested for normality using the Shapiro–Wilk test and, as they were non-normally distributed, were summarized as medians and interquartile ranges (IQRs).

To assess changes in participants' knowledge over time, the Friedman test for repeated measures was used, with post hoc pairwise comparisons using the Wilcoxon signed-rank test and Bonferroni adjustment for multiple comparisons. Effect sizes were reported alongside *p*-values: Kendall's W (W) for the overall Friedman test and the rank-biserial correlation (r) for each pairwise comparison, interpreted using conventional thresholds (approximately 0.1 for small, 0.3 for medium, and 0.5 for large). A two-sided *p*-value below 0.05 was considered statistically significant.

## 3 Results

### 3.1 Collaboration assessment and joint One Health Roadmap development

A total of 70 participants took part in the exercise; their organizational affiliations are shown in Table 1. Based on institutional affiliation, 40.0% represented human health, 27.2% environmental health, and 15.7% animal health.

**Table 1:** Distribution of participants by sector and institution.

Sector	Institution	Participants, n (%)
Human health	NCDC	17 (24.3)
	MoH	11 (15.7)
Animal health	NCAH	11 (15.7)
Environmental health	ESA	10 (14.3)
	MoE	9 (12.9)
Multisectoral control body	FDCC	6 (8.6)
Academia	Faculty of Medicine	3 (4.3)
	Faculty of Veterinary Medicine	2 (2.9)
International organization	WHO	1 (1.4)

NCDC, National Center for Disease Control; MoH, Ministry of Health; NCAH, National Center for Animal Health; ESA, Environmental Sanitation Affairs; MoE, Ministry of Environment; FDCC, Food and Drug Control Center; WHO, World Health Organization.

The structured exercise revealed limited collaboration across most domains. Five technical areas technical-level coordination, finance, laboratory capacity, education and training, and emergency funding received a score of zero across all four scenarios. Stronger collaboration was evident in high- and local-level coordination, legislation and regulation, stakeholders communication, and human resources (scores 3–4; Table 2). During the plenary session, groups presented their findings and discussed lessons learned, underscoring the need for stronger mechanisms to shift from reactive, outbreak-driven collaboration to sustained, institutionalized One Health coordination.

**Table 2:**
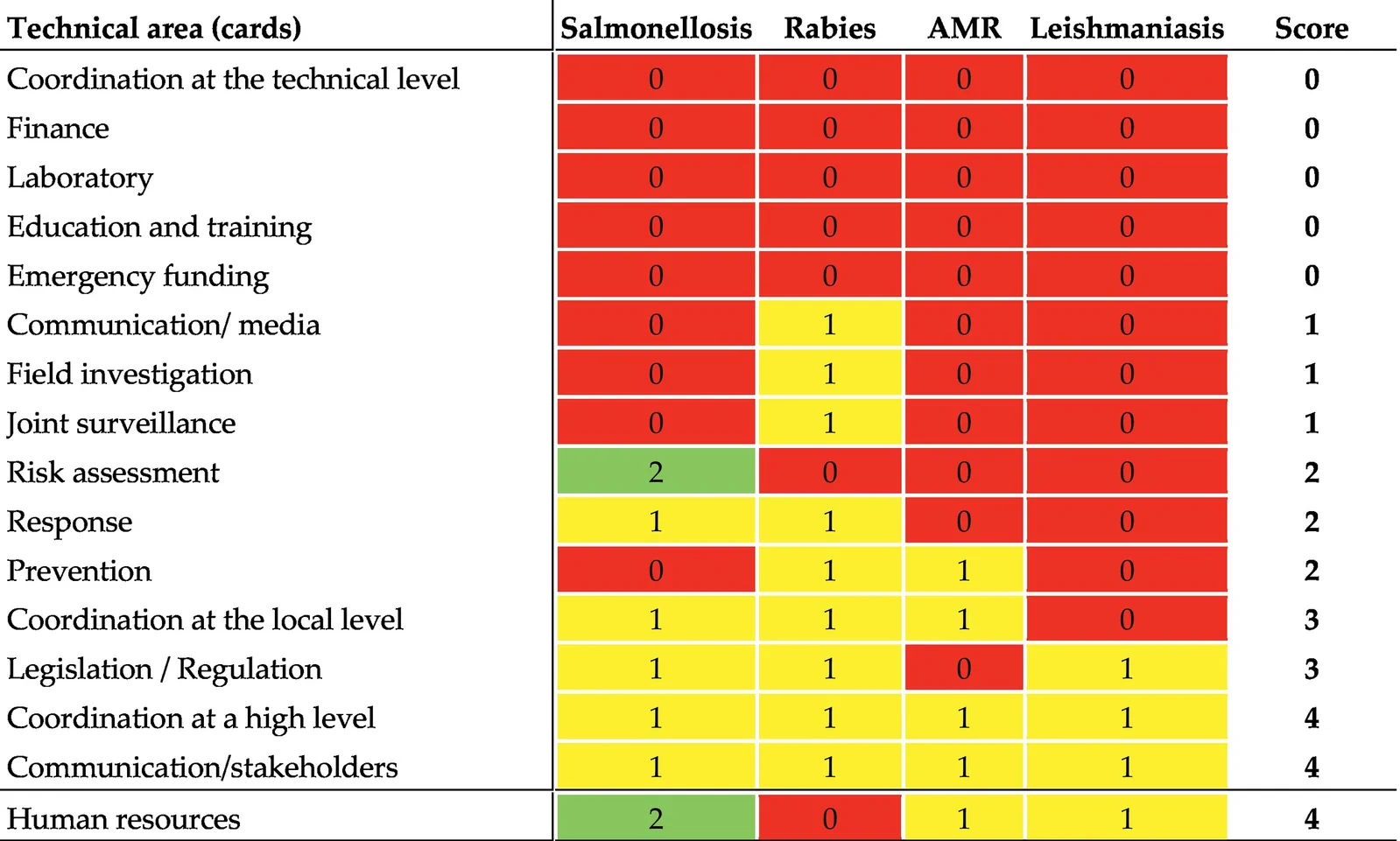
Assessment of collaboration levels across 16 key technical areas

For each hazard, the performance of the collaboration between the human health and the animal health sectors is color-coded: green for “good collaboration”, yellow for “some collaboration”, and red for “collaboration needing improvement”. The score uses a semi-quantitative scale (2 points for a green card, 1 for a yellow card and 0 for a red card). AMR: Antimicrobial Resistance.

### 3.2 Bridges along the road to One Health — IHR/PVS crossroads

After setting the scene through presentations from the relevant sectors in the first session and assessing the current level of cross-sectoral collaboration in the second. During the third session, participants used the cards from the collaboration assessment to plot collaboration levels onto the IHR–PVS matrix, matching them with the corresponding indicators of the IHR MEF and the PVS evaluation. This visual mapping identified points of convergence between the human and animal health systems and overarching patterns of collaboration gaps. A plenary analysis showed clear clusters of gaps and demonstrated that most gaps were systemic rather than disease-specific, revealing five technical domains with the most substantial gaps. To structure the subsequent sessions, participants were organized into Technical Area Working Groups (TAWGs).

**TAWG 1:** Coordination (central, local, technical levels), Legislation, and Finance.

**TAWG 2:** Surveillance, Laboratory, and Risk Assessment.

**TAWG 3:** Field Investigation and Emergency Response.

**TAWG 4:** Prevention and Risk Communication (media and stakeholders), with human resources, education, and training treated as cross-cutting themes addressed by all groups.

Each group extracted the main gaps and recommendations within its assigned technical areas, focusing on opportunities to strengthen cross-sectoral coordination. These outputs provided the foundation for roadmap development.

**Fig. 1:**
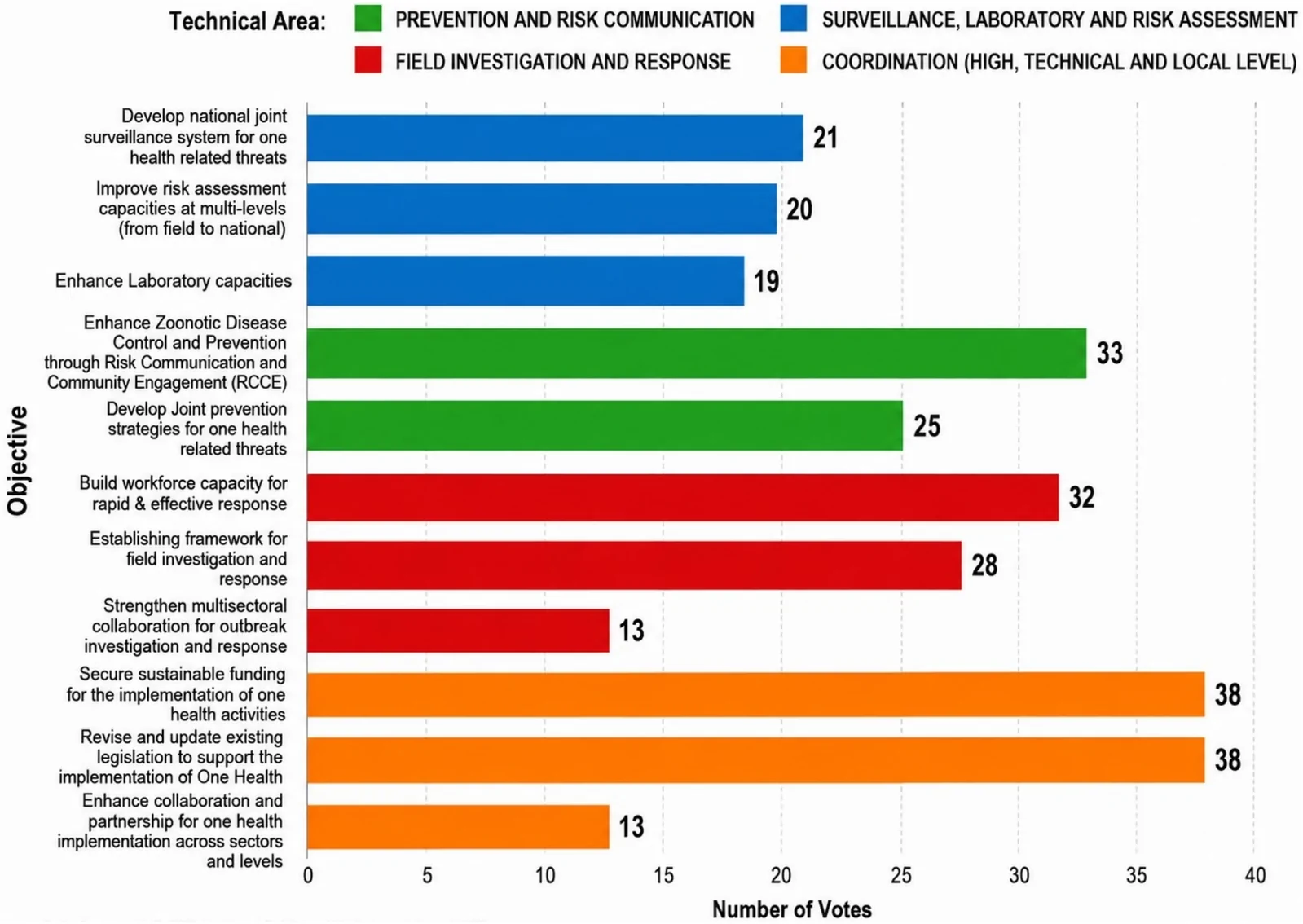
Priority objectives of the One Health Roadmap, by technical area, as identified in the National Bridging Workshop. Numbers refer to the number of votes for each objective as a priority. Total number of used stickers was 273 of 350.

Participants developed the joint NBW One Health Roadmap, comprising 11 objectives and 26 activities to be implemented jointly to manage health threats at the human–animal–environment interface and advance health security in Libya through the One Health operationalization. A participatory ranking exercise using 5 stickers per particiapant to identify the priority objectives to be addressed first. The priority objectives with the most votes (38 each) were to secure sustainable funding for One Health activities and to revise and update existing legislation to integrate One Health into the national framework (laws, executive regulations, and decisions) (Fig. 1), followed by enhancing zoonotic-disease control and prevention through RCCE (33 votes) and workforce capacity building for rapid and effective response (32 votes). The lowest scores (13 each) were attributed to enhancing collaboration and partnerships for One Health implementation across sectors and levels, and to strengthening multisectoral collaboration for outbreak investigation and response.

Table 3, Table 4 summarize the 26 activities by level of difficulty and potential impact. Half (50%) were of medium difficulty, 27% low, and 23% high; most (80.7%) were rated as high impact, and 19.2% as moderate impact.

**Table 3:** The One Health Roadmap: 11 objectives and 26 activities by technical area, with an indicative timeline, difficulty, and impact.

Objective/activity	Timeline	Diff.	Impact
Coordination, legislation, and finance			
Objective 1. Enhance collaboration and partnership for One Health implementation across sectors and levels			votes: 13
1.1 Enhance cooperation among sectors in regions and municipalities	Apr–Oct 2025	++	+++
1.2 Improve coordination and cooperation between technical experts and specialists	Apr–Oct 2025	+	+++
1.3 Develop a national One Health strategy/action plan	Apr–Dec 2025	+	+++
Objective 2. Secure sustainable funding for the implementation of One Health activities			votes: 38
2.1 Advocate for the allocation of financial resources for One Health activities	Sep 2025–Mar 2026	+++	+++
2.2 Develop and implement a cross-sectoral cost-sharing framework	Sep 2025–Mar 2026	+++	++
Objective 3. Revise and update legislation (laws, regulations, decisions) to support One Health			votes: 38
3.1 Activate and operationalize the One Health Memorandum of Understanding	Apr–Dec 2025	+	+++
3.2 Update legislation to incorporate the One Health concept	Jun 2025–Sep 2026	++	++
3.3 Build awareness of legal requirements (e.g., IHR) for decision-makers and workforce	Jun 2025–Sep 2026	++	++

Field investigation and response			
Objective 4. Establish a framework for field investigation and response			
4.1 Develop a One Health national all-hazard preparedness and response plan	Apr–Dec 2025	++	+++
4.2 Develop standardized field investigation protocols for zoonotic diseases	Apr–Dec 2025	+	+++
4.3 Conduct joint simulation exercises for emergency response	Jun 2025–Sep 2026	++	+++
Objective 5. Build workforce capacity for rapid and effective response			votes: 32
5.1 Establish One Health Rapid Response Teams (RRTs)	Apr–Dec 2025	+	+++
5.2 Train RRTs on One Health threats	Jun 2025–Sep 2026	++	+++
5.3 Establish a One Health field epidemiology training and mentorship program	Q4 2026	+++	+++
Objective 6. Strengthen multisectoral collaboration for outbreak investigation and response			votes: 13
6.1 Create a national database for field-investigation data sharing	Jun 2025–Sep 2026	+	++
6.2 Establish a unified incident command system and integrate a One Health PHEOC	Jun 2026–Sep 2027	++	+++
6.3 Organize joint training in incident management systems	Jun 2026–Sep 2027	++	+++

Surveillance, laboratory, and risk assessment			
Objective 7. Enhance laboratory capacities			
7.1 Establish a laboratory management system	Jun 2026–Sep 2027	++	+++
Objective 8. Develop a national joint surveillance system for One Health threats (human, animal, environment)			
8.1 Develop an integrated disease-surveillance joint framework (notifiable/zoonotic, AMR, vector- and food-borne)	Jun 2026–Sep 2028	+++	+++
8.2 Establish a data-sharing mechanism between all sectors	Jun 2025–Sep 2026	++	+++
Objective 9. Improve risk-assessment capacities at multiple levels (field to national)			
9.1 Establish a joint risk-assessment program	Jun 2025–Sep 2026	+++	+++

Prevention and risk communication			
Objective 10. Develop joint prevention strategies for One Health threats (human, animal, environment)			
10.1 Enhance prevention strategies	Jun 2025–Sep 2026	++	+++
10.2 Health promotion (targeted, culturally sensitive campaigns)	Jun 2025–Sep 2027	++	+++
10.3 Develop a joint risk-communication system between sectors	Jun 2025–Sep 2027	++	+++
Objective 11. Enhance zoonotic-disease control and prevention through risk communication and community engagement (RCCE)			votes: 33
11.1 Develop joint immunization campaigns	Jun 2025–Sep 2027	+++	+++
11.2 List all zoonotic diseases and prepare adapted RCCE materials	Jun 2027 - Dec 2027	+	++

Difficulty of implementation: + low, ++ moderate, +++ very difficult. Impact: + low, ++ moderate, +++ high. Priority votes are the participatory-ranking scores reported in the text (blank where the objective was not among the ranked priorities). The full roadmap, including responsible bodies and detailed process steps for each activity, is provided in Supplementary File S1 (Table S1).

**Table 4:** Number of roadmap activities by difficulty and impact, as identified in the National Bridging Workshop.

Level	Activities by level of difficulty, n (%)	Activities by level of impact, n (%)
Low	7 (27.0)	0 (0.0)
Medium	13 (50.0)	5 (19.2)
High	6 (23.0)	21 (80.7)
Total	26 (100.0)	26 (100.0)

The final session, led by national counterparts, discussed the way forward. Building on the MoU as a cornerstone of the One Health approach in Libya, participants underscored that effective operationalization depends on establishing a High-Level Steering Committee by prime ministerial decree as well as an active coordination mechanism and technical working group under the leadership of MoH. They agreed that the NBW roadmap would serve as the foundation for the national One Health strategy and action plan, to be developed by the technical working group once established.

### 3.3 Workshop evaluation and perceived impact

The effectiveness of the workshop was assessed through structured evaluation and knowledge testing. Of the 70 attendees, 55 completed the baseline questionnaire (78.6%), 43 (61.4%) completed the immediate post-test, and 24 (34.2%) completed the three-month follow-up. Table 5.

**Table 5:** Demographic and professional characteristics of participants in the learning intervention.

Characteristic	Number (%)
Gender	
Male	35 (63.6)
Female	20 (36.4)
Age category (years)	
25–30	4 (7.3)
31–44	22 (40.0)
45–54	21 (38.2)
55–65	8 (14.5)
Sector	
Human health	22 (40.0)
Animal health	13 (23.6)
Environmental health	13 (23.6)
Other	7 (12.7)
Discipline*	
Surveillance & epidemiology	10 (18.2)
Laboratory	10 (18.2)
Vector control	9 (16.4)
AMR & IPC	8 (14.5)
Food safety	8 (14.5)
Environmental health & climate change	3 (5.5)
Chemical engineering	2 (3.6)
International health regulations	1 (1.8)
Infectious diseases	1 (1.8)
Slaughterhouse	1 (1.8)
Risk communication & community engagement	2 (3.6)
Veterinary quarantine control	1 (1.8)
Aquaculture	1 (1.8)
Veterinary medicine	1 (1.8)
Infection control	1 (1.8)
Ministry of Health International Cooperation Office	1 (1.8)

N.B. Note that some participants had dual affiliations, AMR: Antimicrobial Resistance, IPC: Infection Prevention and Control.

Knowledge scores differed significantly across the three assessments time points (Friedman test, *p* < 0.001; Kendall's W = 0.44), corresponding to a moderate overall effect. Post hoc pairwise comparisons with Bonferroni-adjusted showed that scores were significantly higher at the immediate post-test than at baseline (median 19 (18–20) vs. 16 (13–18); *p* < 0.001; rank-biserial *r* = 0.64, large effect size) and higher at three months than at baseline (median 18.5 (15–20) vs 16 (13–18); *p* = 0.015; *r* = 0.40, medium effect size). The difference between the immediate post-test and the three-month follow-up was not statistically significant. Table 6.

**Table 6:** One Health knowledge scores across assessment time points.

Assessment time point	Min–Max	Median (Q1–Q3)	*P* value
Pre-assessment^a^	7–20	16 (13–18)	<0.001⁎
Post-assessment^b^	13–20	19 (18–20)	
Three-month follow-up^b^	12–20	18.5 (15–20)	

⁎ Significant, Superscript letters indicate statistically significant differences between time points (p < 0.05); values with different superscripts differ significantly (pre-assessment vs post-assessment and pre-assessment vs 3-month follow-up). For the repeated-measures analysis, only participants with complete data across all three assessment time points were included. Of the initial 70 participants, 55 completed the baseline assessment.

**Fig. 2:**
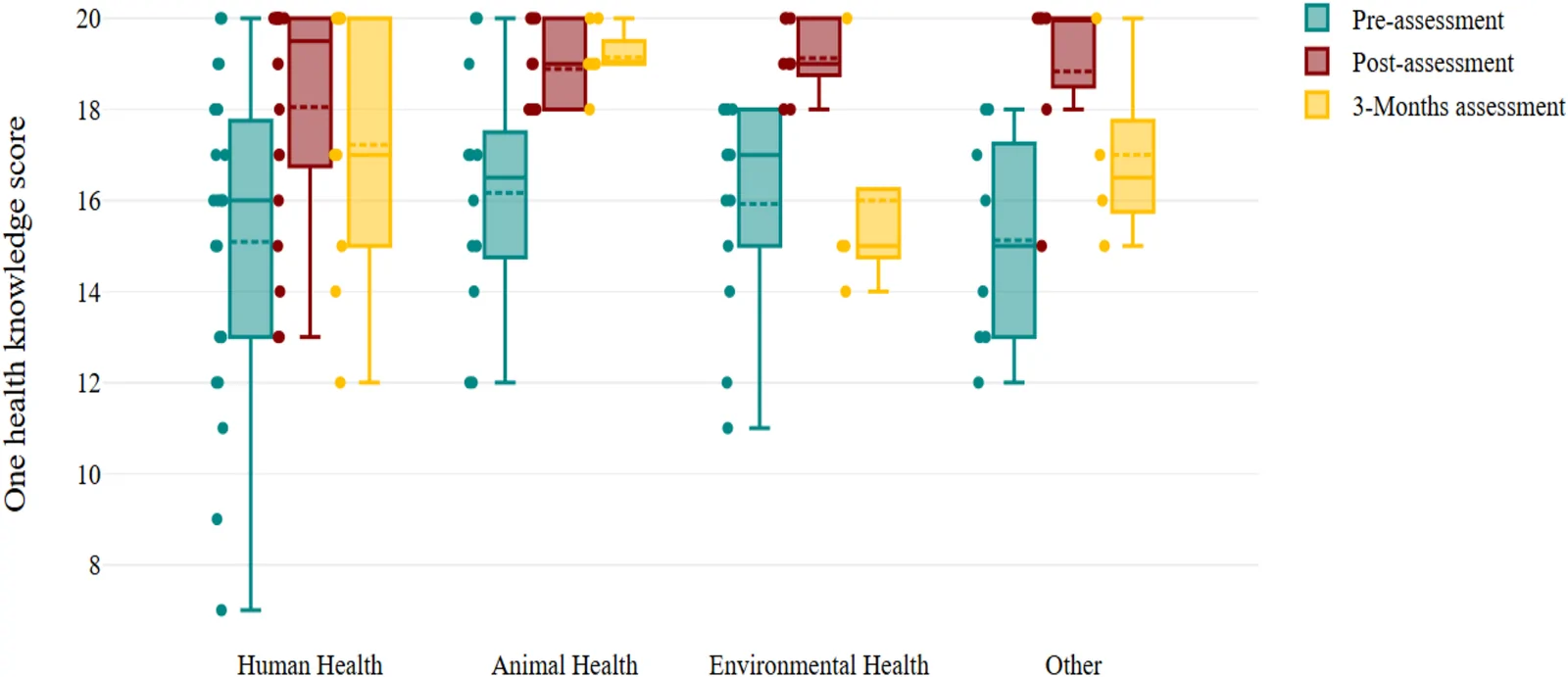
Comparison of One Health knowledge scores by sector across assessment time points.

Across sectors, baseline medians ranged from 15 and 17. Following the workshop, all groups improved, with medians rising to 19–20. At three months, retention varied by sector: participants from the animal health sector maintained high scores (median 19), and those from the human health and other sectors retained improvements relative to baseline (medians 17 and 16.5, respectively), whereas the environmental-health sector showed a decline (median 15). Fig. 2.

## 4 Discussion

This study reports the implementation and outcomes of Libya's first NBW, a milestone toward operationalizing the One Health approach in a fragile context. The workshop achieved broad multisectoral engagement across the human, animal, and environmental health sectors and produced a joint roadmap comprising 11 objectives and 26 activities, offering insights into both the strengths and challenges of implementing One Health in conflict-affected, resource-constrained settings.

The most important finding of the gap assessment was that the lowest-scoring areas including technical-level coordination, finance, education and training, laboratory services, and emergency funding were systemic rather than disease-specific. A pattern confirmed when the gaps were plotted onto the IHR–PVS matrix. Investing in core operational and institutional capacities is likely to yield benefits across multiple hazards simultaneously rather than requiring separate, vertical disease programs. The relatively higher scores for high-level coordination, stakeholder communication, and human resources point to existing strengths that can be leveraged during implementation, and echo observations from other Middle East and North Africa (MENA) settings, where political commitment at senior levels often outpaces technical coordination capacity [[20], [21], [22]]. Using the IHR–PVS matrix as a visual tool to surface these shortfalls was a methodological strength, and integrating JEE, PVS, and internal JEE findings with participant expertise gave the roadmap a defensible evidence base, as has also been reported for the Somalia and Cameroon NBW [[17], [18], [23], [24]].

The collaboration assessment process used health threats that reflected Libya's epidemiological context and the practical need for multisectoral action. The selection of rabies, leishmaniasis, salmonellosis, and AMR was epidemiologically and operationally sound, spanning different transmission pathways and offering varied entry points for cross-sectoral collaboration, consistent with global evidence on the value of country-specific disease prioritization in One Health implementation [[17], [25], [26]].

Although WHO Libya and the ISS facilitated the workshop effectively, the absence of FAO and WOAH representatives owing to security constraints reduced quadripartite presence and may have limited the depth of animal-health and food-security perspectives. This was partly mitigated by the robustness of the NBW methodology jointly developed by WHO, FAO, and WOAH and delivered through standardized materials, including recorded expert contributions. Nonetheless, this limitation underscores the need for adaptive approaches that preserve core methodological principles in challenging operational environments [27].

The prioritization exercise is perhaps the most policy-relevant result. Participants placed the highest priority on financing and legislation rather than on the operational capacities (surveillance, laboratory) that scored poorly in the gap assessment. We interpret this as a recognition that, in Libya's context, a predictable financial base and a clear legal mandate are preconditions for any sustained technical progress. This resonates with a global analysis of 51 NBW roadmaps, which found no single dominant pattern of activities but rather roadmaps tailored to national context [28]; Libya's emphasis on formalizing the legal and financial basis for cooperation reflects a strategic maturity often seen in fragile settings. Notably, coordination gaps, although clearly identified, were not ranked among the top priorities most likely because ad hoc coordination already occurs during outbreaks and has recently been formalized through the MoU. Therefore, participants viewed coordination as a recognized but comparatively tractable challenge relative to financing and legislation.

The decision to establish a high-level steering committee and a technical working group under MoH leadership, and to use the roadmap as the foundation for the national One Health strategy and action plan, signals a clear intent to sustainably operationalize the multisectoral collaboration. Comparable trajectories where NBW roadmaps fed directly into national One Health strategies or the National Action Plan for Health Security have been documented in Kazakhstan, Nigeria, and Pakistan [17], suggesting that Libya's experience may be relevant to other MENA countries facing similar constraints.

Beyond its planning outputs, the NBW functioned as a learning intervention. Knowledge scores were significantly higher immediately after the workshop and remained above baseline for three months. This suggests that improvements in knowledge were maintained among participants who completed the three-month follow-up. Retention nonetheless varied by sector: animal and human health participants generally maintained their gains, whereas environmental health participants declined at three months. This difference is plausibly explained by the smaller number of environmental health respondents at follow-up, differences in baseline familiarity with the material, and fewer opportunities to apply the content in routine practice. The sustained gains suggest that the NBW strengthened participants' understanding of One Health principles and assessment frameworks (IHR MEF and PVS Pathway) and fostered a shared technical vocabulary, a secondary benefit that may support longer-term institutional capacity and preparedness and extend the value of the NBW well beyond the roadmap itself.

## 5 Limitations

Several limitations should be considered. The collaboration assessment relied on participant self-assessment, which may introduce subjectivity and reporting bias, and the short duration of the sessions may have limited in-depth analysis. Some assessment reports were outdated, although national stakeholders' expertise helped to contextualize findings. The under-representation of civil society and non-governmental organizations likely narrowed the perspectives captured; these groups are important for RCCE, and local-level implementation, particularly in fragile or decentralized settings, and their limited involvement may have reduced consideration of community realities in the roadmap. NBW's impact depends on national ownership and sustained political and institutional investment, neither of which is guaranteed.

The substantial loss to follow-up limits the generalizability of the three-month findings and introduces the possibility of attrition bias. Two consequences follow; first, participants who remained engaged enough to complete the three-month assessment may also have been those who learned or retained more, so the retained-knowledge estimates may be somewhat optimistic. Second, the small numbers at follow-up especially within individual sectors, reduce statistical precision and make the sector-level comparisons sensitive to the responses of only a few individuals. The apparent decline among environmental health participants in particular should be read as indicative rather than definitive. The reported knowledge findings should be interpreted with these caveats in mind.

## 6 Conclusion

Libya's NBW brought together representatives from multiple sectors to move from sporadic, outbreak-driven cooperation toward a unified One Health agenda for preparedness and health security at the human–animal–environment interface. Using locally relevant scenarios and the IHR–PVS matrix, participants identified persistent collaboration gaps notably in technical coordination, sustainable financing, laboratory system, workforce development, and emergency funding and translated them into a prioritized roadmap of 11 objectives and 26 activities focused on governance, legislation, and sustainable financing. The roadmap provides a foundation to develop Libya's first National One Health strategy and action plan. Knowledge gains that remained higher than baseline at three months among participants who completed the follow-up assessment, indicate that NBW can also build capacity by reinforcing a shared technical vocabulary and familiarity with essential assessment and planning tools. Next steps should include costing the implementation plan, integrating monitoring indicators, and sustaining high-level political commitment and partner engagement to ensure measurable improvements in preparedness and response.

## Authors contribution

Asma Saidouni: Conceptualization, Questionnaire development, formal analysis, project administration, methodology, writing – original draft, writing – review & editing. Ramy Mohamed Ghazy: Writing – original draft, writing – review & editing, methodology. Ramdhan Mohamed Osman: Writing – review & editing. Abdulaziz Zorgani: Writing – original draft, writing – review & editing. Omar Elahmer: Writing – original draft, writing – review & editing. Hebatalla Abdelmaksoud Abdelmonsef Ahmed: Formal analysis, data visualization, writing – review & editing. Ahmed Elgrari: Writing – review & editing. Guillaume Belot: Questionnaire development, writing – review & editing. Reem Ayad: Questionnaire development, writing – review & editing. Hatem Meslati: Writing – review & editing. Iman Ben Hamza: Writing – review & editing. Hayfa Abraheem Mohammed Alkourbu: Writing – review & editing. Salem Abudher: Writing – review & editing. Maria Grazia Dente: Writing – review & editing. Taher Emahbes: Writing – review & editing. Eszter Horvath: Data analysis, writing – review & editing. Haider Elsaeh: Writing – review & editing. Ahmed Zouiten: Supervision, leadership, writing – review & editing.

## Publisher's note

All claims expressed in this article are solely those of the authors and do not necessarily represent those of their affiliated organizations, or those of the publisher, the editors and the reviewers.

## CRediT authorship contribution statement

**Asma Saidouni:** Writing – review & editing, Writing – original draft, Project administration, Methodology, Formal analysis, Conceptualization. **Ramy Mohamed Ghazy:** Writing – review & editing, Writing – original draft, Methodology. **Ramdhan Mohamed Osman:** Writing – review & editing. **Abdulaziz Zorgani:** Writing – review & editing, Writing – original draft. **Omar Elahmer:** Writing – review & editing, Writing – original draft. **Hebatalla Abdelmaksoud Abdelmonsef Ahmed:** Writing – review & editing, Visualization, Formal analysis. **Ahmed Elgrari:** Writing – review & editing. **Guillaume Belot:** Writing – review & editing, Methodology. **Reem Ayad:** Writing – review & editing, Methodology. **Hatem Meslati:** Writing – review & editing. **Iman Ben Hamza:** Writing – review & editing. **Hayfa Abraheem Mohammed Alkourbu:** Writing – review & editing. **Salem Abudher:** Writing – review & editing. **Taher Emahbes:** Writing – review & editing. **Maria Grazia Dente:** Writing – review & editing. **Eszter Horvath:** Writing – review & editing, Formal analysis. **Haider Elsaeh:** Writing – review & editing. **Ahmed Zouiten:** Writing – review & editing, Supervision.

## Consent for publication

Not applicable.

## Ethics approval and consent to participate

The study protocol was reviewed and approved by the Ethics Committee of the National Center for Disease Control (NCDC), Tripoli, Libya (reference no. NBC: 002.H-26.01). Participation was voluntary, and informed consent was obtained from all participants prior to completion of the questionnaires. Data were collected using unique participant codes without direct personal identifiers and stored securely with access restricted to the study team. The study was conducted in accordance with the ethical principles of the Declaration of Helsinki.

## Funding statement

The authors declare that no financial support was received for the research and/or publication of this article.

## Declaration of competing interest

The authors declare that the research was conducted in the absence of any commercial or financial relationships that could be construed as a potential conflict of interest.

## Data Availability

Data is available upon request by emailing the corresponding author.

## Appendix A Supplementary data

National Bridging Workshop implementation method.
