# Supplementary material for: Strengthening Libya's pandemic preparedness using the National Bridging Workshop One Health Roadmap
Source: One Health. 2026 Aug 19;23:101550. doi: 10.1016/j.onehlt.2026.101550 (PMC13542509; doi:10.1016/j.onehlt.2026.101550)
Supplement: Supplementary file 1 [file mmc1.pdf]

National Bridging Workshop implementation method

The pre-workshop planning

The objectives of the pre-workshop meetings were to familiarize stakeholders with the NBW methodology, agree on the workshop agenda, define participant selection criteria ensuring multisectoral, multidisciplinary, and geographical representation, and jointly develop case scenarios for use during the collaboration gap assessment sessions. Emphasis was placed on national ownership and leadership of the process. Following extensive consultation, a consensus was reached on the selection of rabies, leishmaniasis, salmonellosis, and AMR as case studies. These scenarios were chosen to reflect a broad range of health threats at the human–animal–environment interface, different transmission pathways, and varying opportunities for cross-sectoral collaboration. Rabies and leishmaniasis were prioritized due to their public health importance as zoonotic diseases in Libya (3). Rabies illustrated the relevance of animal population management and waste disposal practices, while leishmaniasis highlighted challenges related to vector control and environmental determinants. AMR was included as a high-priority threat identified in the 2024 Strategic Tool for Analysis of Risks profile, emphasizing the need for integrated surveillance and stewardship across sectors. Salmonellosis was selected as a foodborne disease scenario to assess intersectoral coordination during food safety incidents and outbreak response.

Conduction of the workshop

The workshop was conducted back-to-back with a One Health situation analysis undertaken in collaboration with the Istituto Superiore di Sanità. The workshop followed the standardized NBW methodology and consisted of seven structured sessions guiding participants from gap identification to the development and prioritization of a joint One Health roadmap. The opening session emphasized the importance of multisectoral coordination in addressing health threats at the human–animal–environment interface and highlighted the role of the One Health approach in strengthening national health

security. National and international stakeholders reaffirmed their commitment to supporting One Health operationalization in Libya.

- *Session 1:* introduced the objectives and structure of the workshop through presentations and interactive educational materials. National institutions presented their mandates, capacities, and priority areas, highlighting opportunities for collaboration. Educational videos and interactive quizzes were used to contextualize One Health principles and stimulate engagement.
- *Session 2:* four mixed working groups with balanced sectoral representation were organized. Participants utilized specific case scenarios to evaluate the status of multisectoral collaboration and pinpointed gaps across 16 technical areas through facilitated discussions. During this session, the groups were formed based on the developed case studies. Each group focused on fictional yet locally relevant case studies, including AMR, rabies, salmonellosis, and leishmaniasis, which were created in partnership with national experts. Using color-coded "technical area cards", the groups assessed collaboration in 16 domains, including legislation, surveillance, field investigation, risk assessment, communication, laboratory systems, and emergency response.
- *Session 3:* focused on familiarizing participants with the IHR-MFE and the PVS pathway. Participants used the IHR–PVS matrix to map identified gaps against existing evaluation indicators, enabling visualization of systemic challenges and prioritization of key technical areas.
- *Session 4:* participants regrouped by technical expertise to review findings and recommendations from the JEE, PVS evaluation, and the internal JEE conducted in 2025. Findings from Session 2 were used to contextualize and update recommendations.
- *Session 5:* involved the formulation of specific objectives and joint activities aimed at addressing the identified gaps and strengthening multisectoral collaboration.

- *Session 6*: the objectives were consolidated into a single joint One Health roadmap through plenary discussion and a rapid prioritization exercise based on feasibility, cost-effectiveness, and potential impact.
- *Session 7*: consisted of a final plenary discussion led by national representatives to identify enabling factors for roadmap implementation and outline next steps for operationalizing the One Health approach.
