# Supplementary material for: Strengthening Libya's pandemic preparedness using the National Bridging Workshop One Health Roadmap
Source: One Health. 2026 Aug 19;23:101550. doi: 10.1016/j.onehlt.2026.101550 (PMC13542509; doi:10.1016/j.onehlt.2026.101550)
Supplement: Supplementary file 2 [file mmc2.pdf]

Questions Responses Settings Total points: 2(

Pre test

Form description

General Information

The NBW workshop fosters mutual understanding among sectors, facilitating discussions on collaboration, its strengths and weaknesses, and the formulation of a joint, consensual roadmap. his questionnaire is designed to evaluate the effectiveness of the workshop content in conveying the core principles of the One Health approach. Your feedback will guide us in refining the materials and improving future delivery methods to better support multisectoral engagement. The answers provided in this survey are confidential. The information collected will be compiled for analysis and research purposes and presented in a confidential manner without revealing individuals' names. This measure aims to ensure the privacy of all participants and encourage them to provide honest and candid opinions without reservation. Completing the survey takes approximately 10 minutes. Thank you for dedicating your valuable time to this survey.

1. **Anonymous ID (Number given to you on arrival)**

Short answer

Short answer text

Answer key (0 points)

Required

2. Please indicate the sector in which you work: *

- [ ] Human Health
- [ ] Animal Health
- [ ] Environmental Health
- [ ] other

If other, please specify

Short answer text

3. What is your primary discipline? *

- [ ] Surveillance and epidemiology
- [ ] Laboratory
- [ ] Vector control
- [ ] Food Safety
- [ ] AMR & IPC
- [ ] Risk Communication and Community Engagement
- [ ] Other: ____________

If other, please specify

Short answer text

4. What level do you work at: *

- [ ] National level
- [ ] Subnational level
- [ ] District/Field level

5. In the last three years, did you attend any specific course on One Health? *

- [ ] Yes
- [ ] No
- [ ] I don't recall

General Knowledge

Description (optional)

1. One Health is an integrated, unifying approach that aims to sustainably balance and optimize the health of people, animals, and ecosystems. *

- Correct
- Incorrect
- I don't know

2. The One Health approach recognizes that the health of humans, domestic and wild animals, plants, and the wider environment are closely linked and interdependent. *

- Correct
- Incorrect
- I don't know

3. The One Health approach is necessary because it addresses interconnected health challenges like zoonotic diseases, antimicrobial resistance, food safety, and ecosystem health. *

- Correct
- Incorrect
- I don't know

4. 75% of emerging infectious diseases are zoonotic. *

- Correct
- Incorrect
- I don't know

5. 60% of known human pathogens are zoonotic. *

- Correct
- Incorrect
- I don't know

6. Air pollution contributes to the overall burden of disease and increases the use of antimicrobials, promoting antimicrobial resistance (AMR). *

- Correct
- Incorrect
- I don't know

7. How does vaccination contribute to combating Antimicrobial Resistance (AMR)?

- ◯ Vaccines directly kill antibiotic-resistant bacteria, eliminating the need for antibiotics.
- ◯ Vaccination reduces the incidence of infections, thereby decreasing the need for antibiotics and slowing ...
- ◯ Vaccination has no impact on AMR, as it only targets viral infections.

8. Biodiversity loss disrupts ecosystems, favoring the spread of zoonotic pathogens. *

- ◯ Correct
- ◯ Incorrect
- ◯ I don't know

9. Climate change significantly alters habitats by shifting temperature and precipitation patterns, affecting species distributions and ecosystem interactions. *

- ◯ Correct
- ◯ Incorrect
- ◯ I don't know

10. Non-communicable diseases (NCDs) are the largest disease burden globally and are increasingly affected by climate change. *

- ◯ Correct
- ◯ Incorrect
- ◯ I don't know

11. Plastic pollution poses NO significant threat to terrestrial and marine ecosystems. *

- Correct
- Incorrect
- I don't know

12. Deforestation shifts natural areas to human-dominated areas, increasing the risk of zoonotic diseases. *

- Correct
- Incorrect
- I don't know

13. Joint risk assessment should be conducted frequently to ensure effective collaboration. *

- Correct
- Incorrect
- I don't know

14. Coordination and data sharing are essential during joint investigations of zoonotic outbreaks. *

- ◯ Correct
- ◯ Incorrect
- ◯ I don't know

15. The International Health Regulations (IHR) are a legally binding framework aimed at improving global health security. *

- ◯ Correct
- ◯ Incorrect
- ◯ I don't know

16. Using a One Health approach allows for a better implementation of the IHR (2005) through: *

- ◯ The detection of risk factors in the environment or in animal populations that can be used as sentinels.
- ◯ Developing fragmented response plans that lack coordination and delay control measures.

17. The Joint External Evaluation (JEE) is a mandatory process initiated by WHO *

- ◯ Correct
- ◯ Incorrect
- ◯ I don't know

18. The PVS Pathway is a voluntary, country-driven process aimed at improving compliance of Veterinary Services with international standards. *

- ◯ Correct
- ◯ Incorrect
- ◯ I don't know

19. What types of health challenges does the OHJPA primarily aim to address? *

- ◯ Only non-communicable diseases like diabetes and heart disease
- ◯ Zoonotic diseases, antimicrobial resistance, and environmental health risks.
- ◯ Exclusively mental health issues in urban populations
- ◯ Economic disparities in healthcare access

20. What organizations are part of the One Health Quadripartite? *

- ◯ WHO, UNICEF, FAO, UNEP
- ◯ WHO, WOAH, FAO, UNEP
- ◯ WHO, WOAH, FAO, UNICEF
